# Casing deformation mechanisms of horizontal wells in seismically active zones: a comprehensive analysis

**DOI:** 10.1038/s41598-025-94469-1

**Published:** 2025-03-28

**Authors:** Hu Meng, ShuangJin Zheng, ZhenXin Jiang, XiaoQiong Wang, Yinghao Shen, ShengXuan Zhou, YunChao Pu, HongKui Ge

**Affiliations:** 1 Hubei Key Laboratory of Oil and Gas Drilling and Production Engineering, Yangtze University), Wuhan, 430100 China; 2 School of Petroleum Engineering, Yangtze University, Wuhan, 430100 China; 3 PetroChina Zhejiang Oilfield Company, Hangzhou, 310000 China; 4 Unconventional Oil and Gas Science & Technology Institute, China University of Petroleum (Beijing), Beijing, 102249 China; 5 PetroChina Jilin Oilfield Company, Songyuan, 138000 China; 6 Sinopec International Petroleum Exploration and Production Corporation, Beijing, 100029 China

**Keywords:** Seismicity, Hydraulic fracturing, Fault slip, Casing shear deformation, Strength design, Natural gas, Crude oil

## Abstract

The Sichuan basin’s shale gas fields demonstrate elevated seismic activity, which poses a significant challenge in the development of shale gas. Besides, casing deformation emerges as a prominent concern, leading to substantial disruptions in shale gas production operations. In order to address the issue of casing deformation in seismically active areas, an analysis was conducted on the seismicity and casing deformation. Subsequently, a three-dimensional finite element model was developed to represent the casing-cement sheath-fault-formation assembly. A study was conducted to investigate the mechanism of casing shear deformation caused by fault slip, as well as the corresponding mechanical response of the casing. An investigation was conducted to analyze the influence of drilling and fracturing parameters on casing shear deformation. Additionally, strategies to effectively manage casing shear deformation were proposed. Furthermore, a novel approach to designing casing strength was introduced. The analysis of the data reveals that the Weiyuan, Changning, and Zhaotong shale gas fields exhibit high levels of tectonic stress, notable disparities in horizontal stress, and well-developed fault systems. The aforementioned factors are responsible for an increased occurrence of casing shear deformation and a greater probability of triggering earthquakes. The occurrence of fault slip results in the escalation of casing deformation and stress within a 1-meter vicinity of the fault. The casing Mises stress surpasses the yield strength with relative ease. The inclusion of casing deformation quantity in casing strength design can significantly reduce downhole incidents resulting from casing shear deformation during hydraulic fracturing operations.

## Introduction

The shale gas resources in China exhibit substantial potential for development and have effectively attained commercial production, rendering them a vital natural gas resource^1–3^. Hydraulic fracturing serves as the core technology for shale gas development, but it also poses potential geological hazards, including induced earthquakes^4^. The seismic activity in the vicinity of the shale gas field located in the Sichuan basin has experienced notable amplification and has been thoroughly documented due to the ongoing exploration and development of shale gas^5–11^. Faults within shale gas reservoirs are activated by high pressure fluid with large displacement during hydraulic fracturing, resulting in inducing seismicity^12,13^. Seismicity induced by hydraulic fracturing has been detected through microseismic monitoring and seismic networks. The Sichuan basin and Canada have experienced the highest magnitudes recorded, with ML5.7 on December 18, 2018 and MW4.4 on January 12, 2016, respectively^9,14^. Seismological methods, such as seismicity spatio-temporal evolution, b-value and focal mechanism, have predominantly been employed to study the seismicity induced by shale gas production in Sichuan basin^6,14–16^. It can be seen from the induced seismic characteristics that there is a strong correlation between induced seismic activity and industrial activity in time and space^17^.

The Weiyuan shale gas field (WYSGF), Changning shale gas field (CNSGF) and Zhaotong shale gas field (ZTSGF) located in the Sichuan basin demonstrate both a significant level of seismic activity and notable casing shear deformation in shale gas horizontal wells. These phenomena have resulted in failures during fracturing operations and production challenges. This viewpoint has been proved by multi-arm caliper measurement and the lead mold. In 2019, Xi determined that the casing shear deformation rate in the WYSGF-CNSGF reached 61.7%^18^. In 2017, Chen proposed that shear deformation predominantly occurs in the casing near natural fractures or faults during hydraulic fracturing and established a model to calculate fault slip distance using the focal mechanism parameters^19,20^. The WYSGF, CNSGF, and ZTSGF exhibit the presence of natural fractures and faults, which renders them prone to activation when hydraulic fracturing is conducted. Consequently, this activation can lead to casing shear deformation^21–23^. Several finite element models have been established to study the mechanical behavior of casing at different slip distances, but the quantitative description of slip distances was neglected^24–26^. Furthermore, the development of two-dimensional models allowed for the calculation of slip distances without taking into account the influence of the casing^27–29^. In 2021, Tong revealed the reasons for frequent casing shear deformation in the WYSGF and highlighted the need for integrated studies^30,31^. Existing research often investigates casing deformation and fault slip in isolation, neglecting their interaction. The calculation of casing stress and deformation heavily relies on accurately determining the fault slip distance.

The characteristics of seismicity and casing deformation in shale gas field were analyzed, and the relationship among seismicity, casing deformation, structural features, stress difference, and fracture development degree was explored. Furthermore, this study addresses these limitations by developing a three-dimensional geomechanical model that integrates casing, cement sheath, fault, and formation. The model incorporates full-scale faults and considers sliding interactions and fracturing fluid effects, providing a comprehensive framework to analyze casing mechanical behavior under fault slip. Additionally, the research investigated drilling and hydraulic fracturing impacts on casing deformation and proposes mitigation strategies. By bridging the gap in understanding casing shear deformation mechanisms and fault slip interactions, this study offers valuable insights. It provides a robust framework for analyzing and mitigating casing shear deformation, enhancing the safety and efficiency of hydraulic fracturing operations in seismically active zones like the Sichuan basin.

## Overview of seismicity and casing deformation in shale gas producing areas

**Fig. 1:**
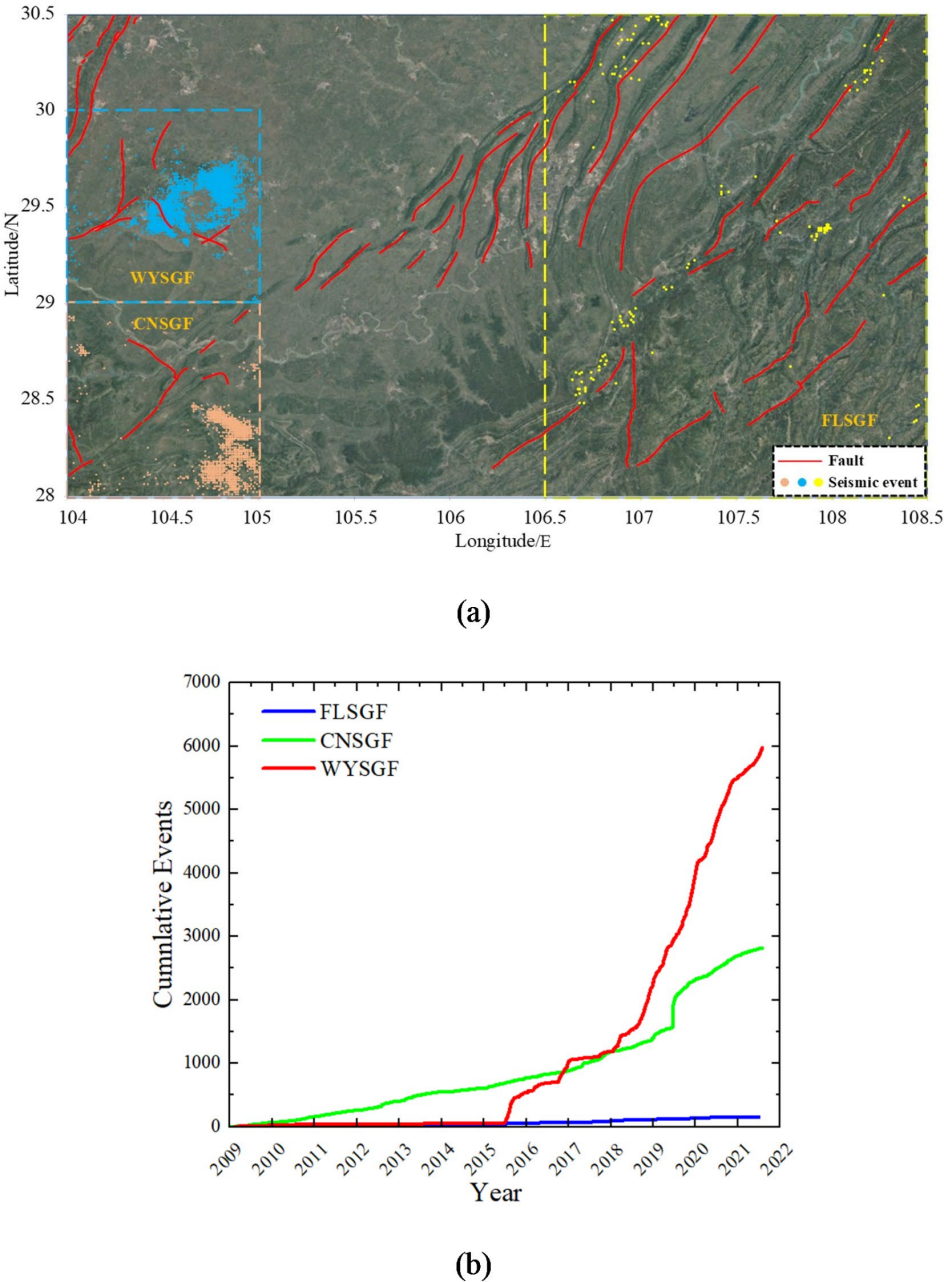
Distribution of seismic events. (**a**) Spatial distribution, (Temporal distribution)

Natural gas is widely available in China’s Sichuan basin, which also has the greatest shale gas reserves in the nation. The WYSGF, CNSGF, ZTSGF, and Fuling shale gas field (FLSGF) have undergone commercial development. Shale gas has been the main driver of natural gas growth in China, making it the second-largest shale gas producer in the world. There is a lot of seismic activity in these shale gas fields^32–34^. The seismic catalogs from January 1, 2009 to September 1, 2021 were analyzed in these regions to determine the regional seismic frequency. In seismic analysis, the combination of CNSGF and ZTSGF is commonly referred to as CNSGF due to their close proximity. The Jianwu syncline is developed in CNSGF. The dip angle of its two wings is 5°-10°, and the core stratum is nearly horizontal, connecting with Changning anticline to the north^35^. In contrast, the main geological features of WYSGF are Weiyuan large dome anticline structure in the direction of NE-SW^36^. Furthermore, the FLSGF is located at the junction of Shizhu syncline, Fangdoushan syncline and Wanxian syncline^37^. There are some faults of different scale in the three study areas. Figure 1 depicts the spatio-temporal distribution of seismic events with M_L_≥1 and a depth of 1–5 km in three regions: WYSGF (with coordinates ranging from E104° to E105° and N29° to N30°), CNSGF (with coordinates ranging from E104° to E105° and N28° to N29°), and FLSGF (with coordinates ranging from E106.5° to E108.5° and N28° to N30.5°). Without considering the difference in seismic network density, the WYSGF has the highest number of seismic events, followed by CNSGF and FLSGF. Seismic events in WYSGF began to increase in 2015, whereas those in CNSGF began to surge in 2010. The seismic events in the FLSGF exhibit a linear increase over time, with FLSGF demonstrating the lowest occurrence of seismic events within this particular fields.

Furthermore, an analysis was conducted on the tectonic structure, in-situ stress, and casing deformation rate of these blocks using seismic data, Kaiser experiment for acoustic emission, small scale fracturing, and statistics data^38–41^. The findings, as shown in Table 1, demonstrate that CNSGF and ZTSGF display a higher stress difference, followed by WYSGF, while FLSGF exhibits the lowest stress difference. Besides, it is found that the regional stress difference is small in the anticline due to the relatively high tectonic uplift and relatively small buried depth, while the stress difference is large in the anticline slope and syncline slope due to the compression tectonic state. The determination of in-situ stress magnitude is strongly influenced by factors such as formation depth, regional tectonic morphology, and tectonic stress strength. As indicated in Table 1, casing deformation primarily occurs in the anticline slope and syncline slope, where there is a significant stress difference and development of natural fractures and faults. The FLSGF structure can be classified as a tension-compression anticline, characterized by stress release and a relatively small stress difference. Despite the development of natural fractures, the rate of casing deformation remains significantly low. In addition, the casing deformation rate of different blocks (Huangjinba, Zijinba, Taiyang, Dazhai) in ZTSGF is quite different, and the blocks with large stress difference and developed natural fractures often have higher casing deformation rate. The blocks WYSGF, CNSGF, and ZTSGF exhibit significant seismic activity, accompanied by a notable rate of casing deformation. The casing deformation rates of these blocks have reached 57.7%, 31.9%, and 22.5% by April 2019. It is worth noting that the FLSGF exhibits low seismic activity and a low casing deformation rate.

**Table 1:** Characteristics of shale gas fields.

Shale gas field	Horizontal stress difference(MPa)	Tectonic position	Tectonic stress field state	Stress direction	Degree of fracture development	Casing deformation rate(%)
WYSGF	9 ~ 15	syncline slope	main compression	NWW-SEE	local development	57.7
CNSGF	21.4 ~ 22.3	syncline slope	compression	NWW-SEE	local development	31.9
Huangjinba block of ZTSGF	18 ~ 30	synclinal axis and syncline slop	compression-twist	NWW-SEE	relative development	33.3
Zijinba block of ZTSGF	12.5 ~ 25	syncline slope	compression-twist	NWW-SEE/NNE-SSW	development	42.9
Taiyang block of ZTSGF	4 ~ 16	anticlinal uplift	compression-twist	NNE-SSW	relative development	16.3
Dazhai block of ZTSGF	30	syncline slope	compression	NNE-SSW	relative development	26.4
FLSGF	3 ~ 6	box-shaped anticline	main compression	Near EW	development	low

**Fig. 2:**
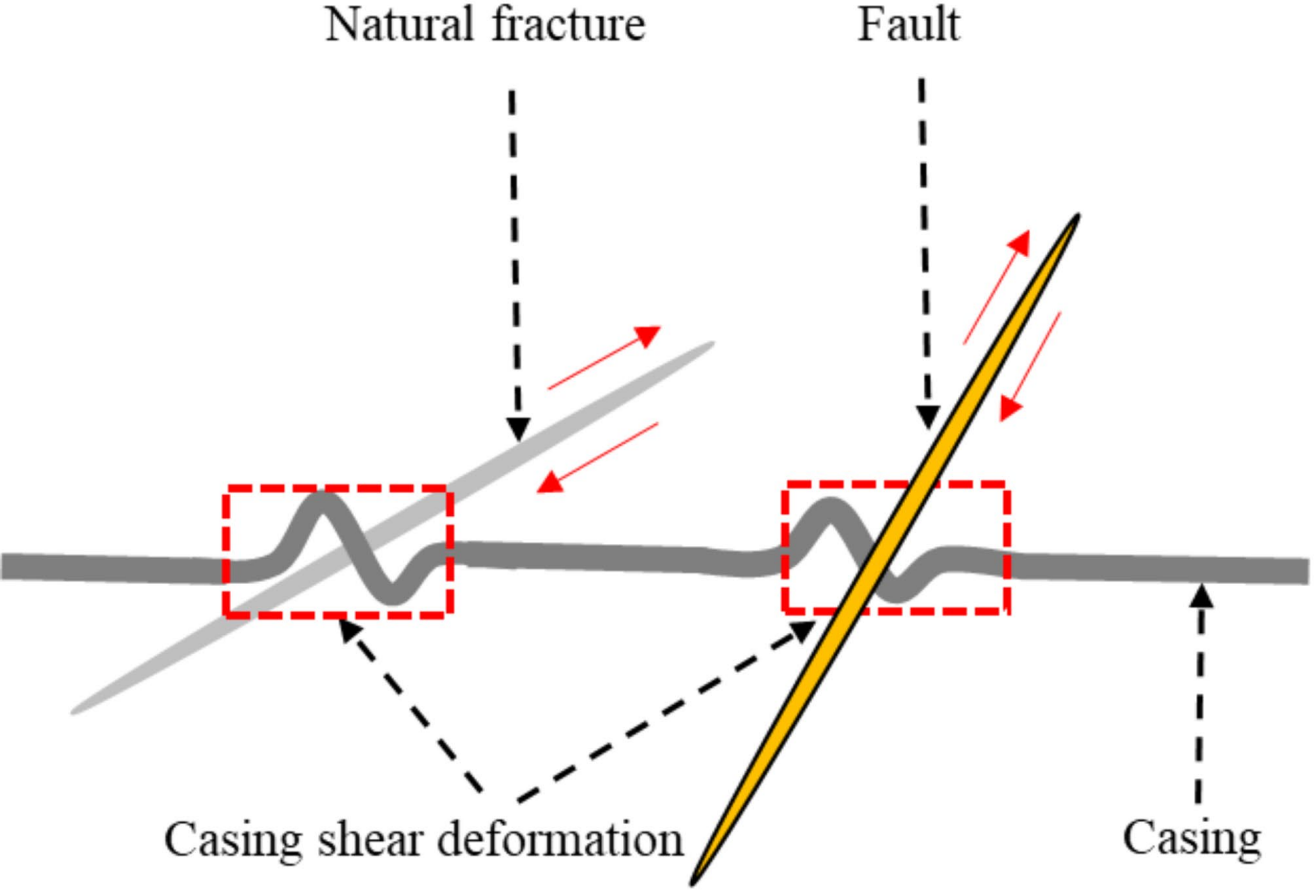
Schematic diagram of casing shear deformation.

The casing deformation shape can be determined by utilizing multi-arm caliper logging. This technique serves as a foundation for studying the mechanism behind casing deformation. The predominant forms of casing deformation observed in WYSGF, CNSGF, and ZTSGF are shear deformation and extrusion deformation^18^. The shale of Longmaxi formation in Sichuan basin has experienced multi-stage tectonic movement and developed multi-stage, multi-scale, multi-type and multi-direction natural fracture system, which leads to many events such as well loss and stuck during drilling^42–44^. Casing shear deformation is the most serious in Sichuan Basin, and the activation of faults, natural fractures and bedding is the main cause of casing deformation during hydraulic fracturing^19,22,26,45^, as shown in Fig. 2. The biggest problem caused by casing shear deformation is that the reduction of casing inner diameter, which may eventually cause the downhole tools to be unable to pass through the deformed casing section and affect the fracturing operations. The analysis of casing deformation points in WYSGF, CNSGF, and ZTSGF reveals that a significant majority, specifically over 60%, are situated in close proximity to faults, natural fractures, and bedding. During hydraulic fracturing, these natural weak surfaces are activated under the action of large downhole construction pressures. This finding has been verified through the utilization of microseismic monitoring techniques and fracture ant body examination^46^.

## Geomechanical model for casing deformation

During the hydraulic fracturing process, the fracturing fluid is introduced into the fault via hydraulic fractures, natural fractures, bedding, and cement sheath^47^. When the exerted on the surface of the fault reaches a critical threshold, the fault undergoes slipping. As a result, the casing experiences shear failure due to the displacement load caused by the fault. A geomechanical model was developed in order to simulate the process of casing shear deformation and examine the correlation between fault slip and casing failure. The objective of this study was to investigate the mechanism underlying casing shear deformation and understand relationship between fault slip and casing failure.

### Description of model

**Fig. 3:**
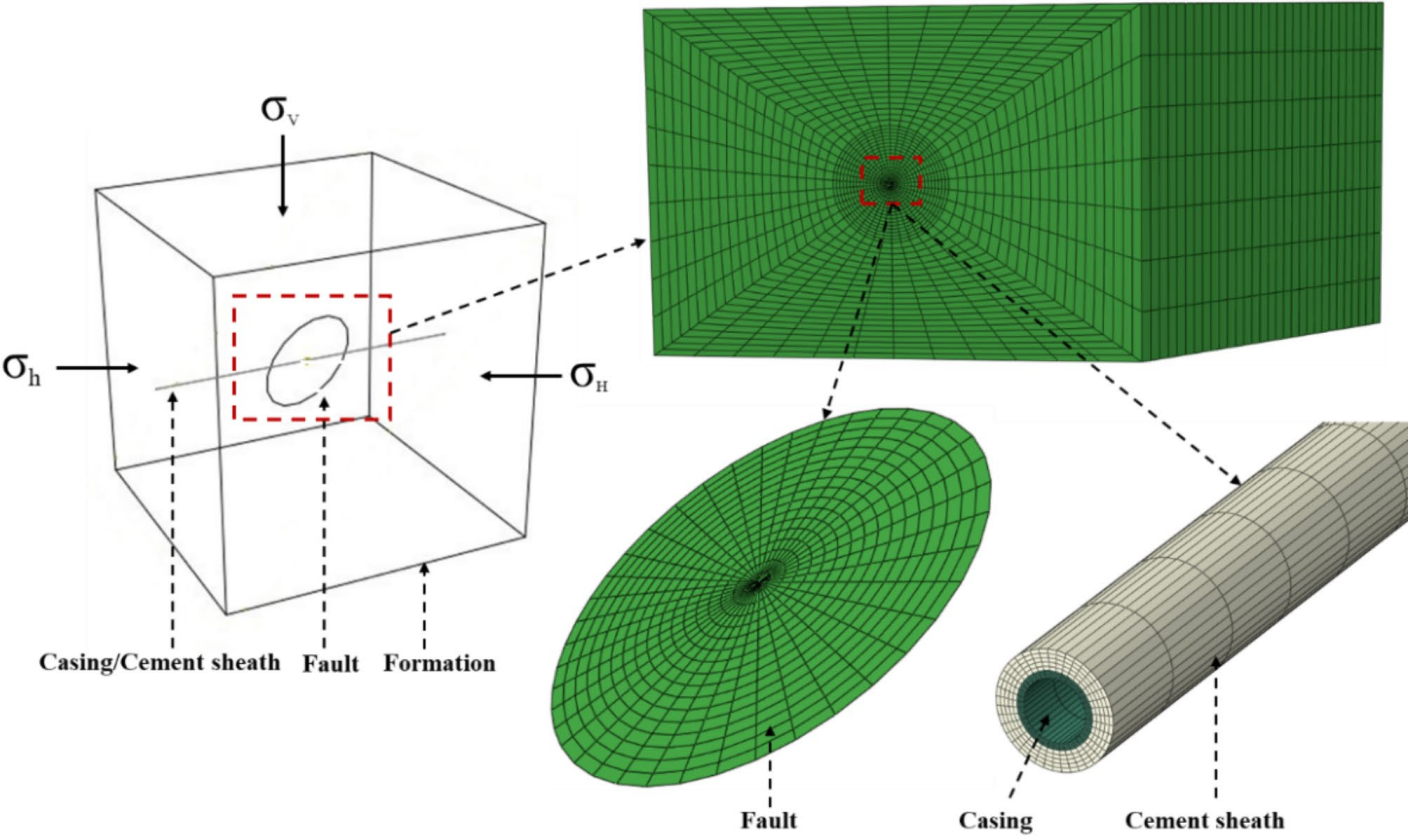
Geomechanical model of casing-cement sheath-fault-formation assembly.

Circular and rectangular fault are frequently employed in fault simulation. In shale gas drilling, the wellbore frequently encounters natural fractures and small faults^23^. While large faults are easily identified and can be avoided, smaller faults pose a challenge. To accurately simulate fault slip, a circular fault, which is better suited for representing small faults, was chosen for modeling purposes^20,48^. The simulation of fault slip induced by fluid injection into a rectangular fault has been conducted by Rutqvist and Hu^49,50^. Nevertheless, the aforementioned studies failed to account for the resistance exerted by the surrounding formation in the vicinity of the fault tip. The impact of the fault tip was taken into account, and a geomechanical model was developed to represent the casing-cement sheath-fault-formation assembly, accurately reflecting the interaction between the wellbore and the fault. The model is depicted in Fig. 3. The position of the casing relative to the fault can be adjusted according to the actual situation, and the casing at the center of the fault is just one of those situations. Besides, other types of faults can also be simulated based on the method used to establish the model.

This model links fault activation and casing deformation. The fault activation induced by fluid injection is classified into three categories: (1) the direct entry of injected fluids into fault; (2) the effect of poroelastic stress; (3) the effect of aseismic slip. The direct entry of fracturing fluids into fault is the main way leading to its sliding during hydraulic fracturing^31,33^. In the process of fracturing fluid injection, when the hydraulic fracture is connected to the fault and the fracturing fluid enters the fault, the fluid pressure in the fault increases to a critical value and the fault begins to slip.

A quasi-static analysis was chosen to disregard the dynamic effect of the fault^51^. In this analysis, the fault is assumed to initially remain stable, then it slips under constant fluid pressure, and finally stabilizes again. The stress equilibrium equation for the solid element can be expressed as follows:

1 $$\:{\sigma\:}_{ij,j}+{F}_{i}=0$$

Where $\:{\sigma\:}_{ij}$ is the stress tensor of the element, *i* = 1,2,3, *j* = 1,2,3, Pa; $\:{\sigma\:}_{ij,j}$ is the partial derivative of $\:{\sigma\:}_{ij}$, N/m^3^; $\:{F}_{i}$ is the volume force in all directions of the element, N/m^3^.

The fault was modeled as the contact surfaces, which transmit the shear stress and normal stress. The Coulomb friction law was applied to the two fault faces, causing them to slide when the tangential stress exceeds the friction strength. The friction strength of the fault is as follows:

2 $$\:{\tau\:}_{c}=\mu\:({\sigma\:}_{n}-P)$$

Where $\:{\tau\:}_{c}$ is the friction strength of the fault, Pa; $\:{\sigma\:}_{n}$ is the normal stress of the fault, Pa; $\:\mu\:$ is the friction coefficient of the fault; $\:P$ is the fluid pressure in the fault, Pa.

The casing, cement sheath and formation were established separately according to the field parameters. The Coulomb friction contact was established between the casing and the cement sheath, as well as between the cement sheath and the wellbore, with a friction coefficient of 0.6 for both interfaces^24^. The finite element software ABAQUS was employed to solve the model^52^. In order to streamline the calculation process, several assumptions were taken into account. These include: (1) disregarding the cementation strength of the fault; (2) assuming a uniform distribution of fluid pressure along the fault and casing; and (3) neglecting the matrix permeability and the flow process.

### Parameters setting and boundary conditions

**Fig. 4:**
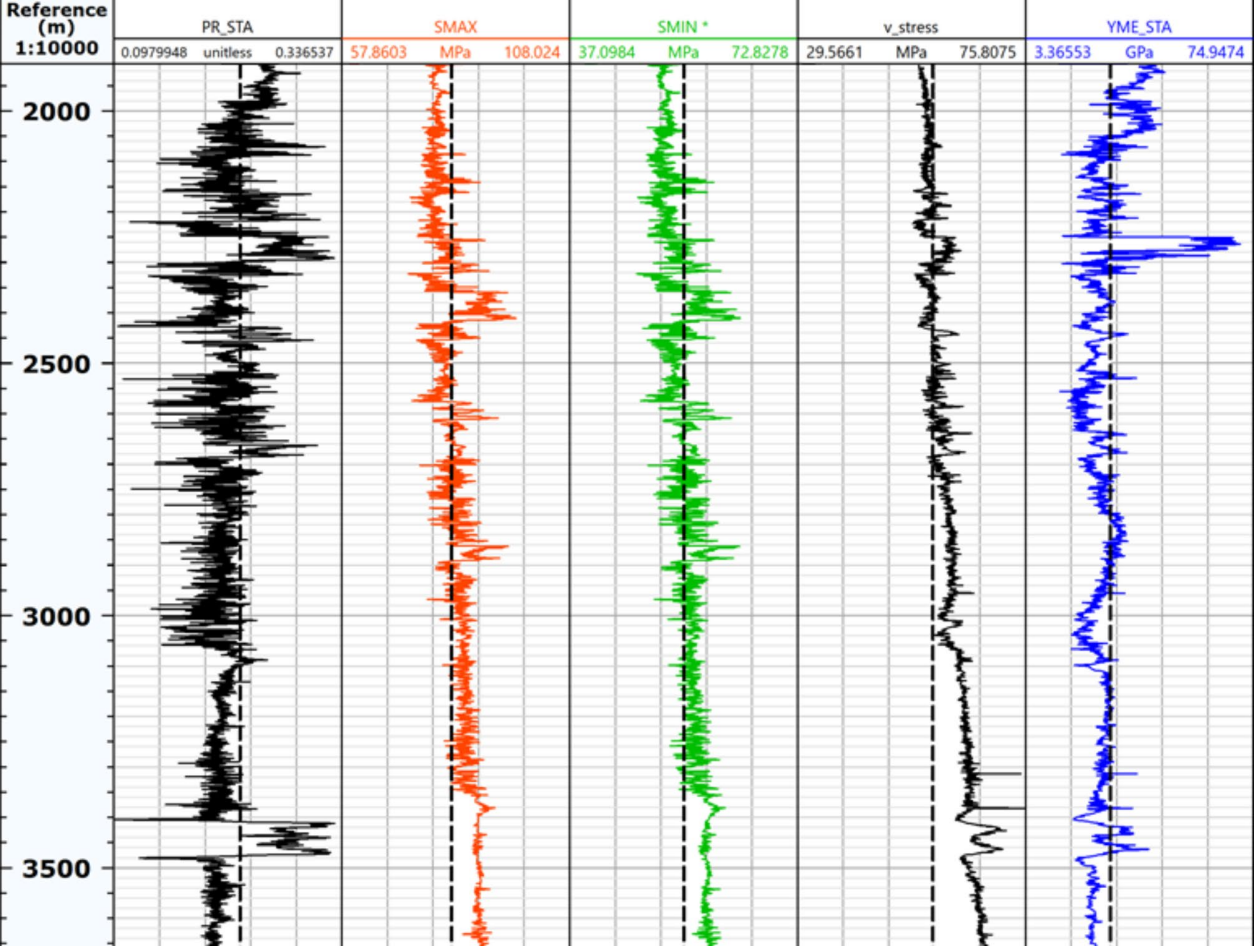
The in-situ stress analysis by one-dimensional geomechanics.

The primary fault types observed in the shale gas fields of the Sichuan basin include reverse faults and strike-slip faults. The model parameters are obtained based on rock mechanics experiments, ant body analysis, and geomechanics analysis of a horizontal well. The in-situ stress and formation elastic parameters are shown in Fig. 4. Taking the strike-slip fault as an example, the average maximum horizontal stress, minimum horizontal stress, and vertical stress are 82, 55, and 57 MPa, respectively. The fluid pressure within the fault is 65 MPa, while the fault’s length, dip angle, and friction coefficient are 100 m, 45°, and 0.6, respectively. The elastic constitutive law was employed to model the formation and cement sheath, while the casing was represented using the bilinear constitutive law. Table 2 presents the geometric and mechanical parameters of the formation, cement sheath, and casing. To mitigate boundary effects, the model dimensions are 400 m ×400 m × 400 m. All parts of the model were discretized using the C3D8R element with the free mesh technique. Grid refinement was implemented in the vicinity of the fault in order to accommodate significant deformations. The quantities of elements required for the formation, cement sheath, and casing are 45,600, 16,000, and 50,400, respectively.

The outer boundary of the model was subjected to the normal displacement constraints, whereas the fault surface and casing inner wall experienced the application of fluid pressure. The initial in-situ stress was applied by predefined field method, defined by the keywords (*PREDEFINED FIELDS), and the geostatic step was used to balance the in-situ stress and external load to get an initial state with a displacement of 0.

**Table 2:** Parameters of model.

Category	Inner diameter (mm)	Outer diameter (mm)	Young’s modulus (GPa)	Poisson’s ratio	Yield strength (MPa)	Tangent modulus (MPa)
Formation	215.9	–	30	0.23	–	–
Cement sheath	139.7	215.9	10	0.17	–	–
Casing	114.3	139.7	210	0.3	758	2000

## Results and discussion

### Casing response to fault slip

**Fig. 5:**
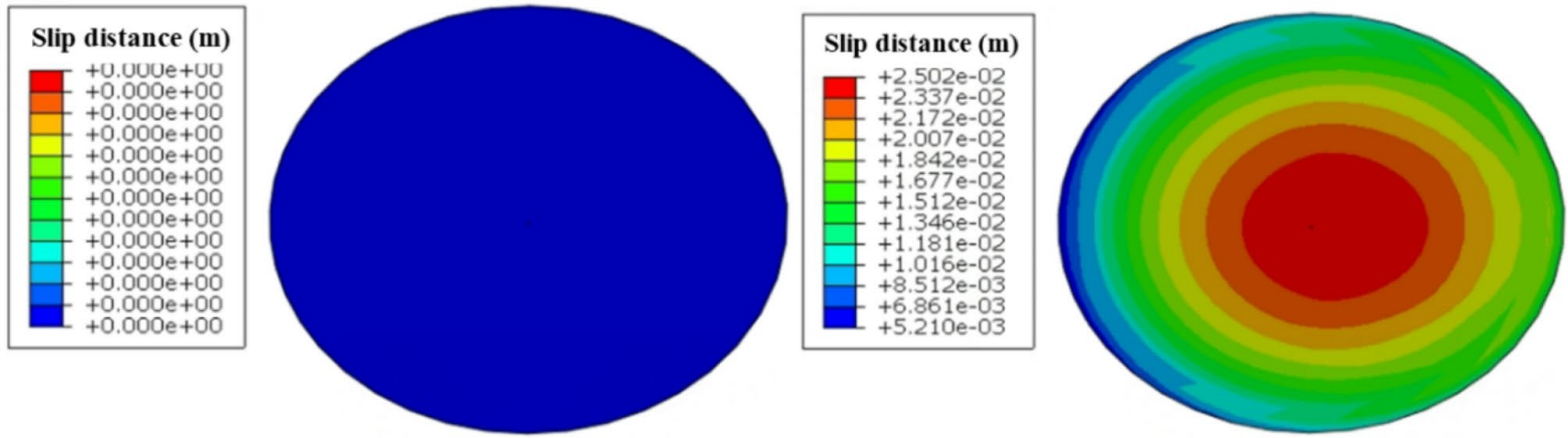
Characteristics of fault slip. (**a**) Slip distance of fault before slipping (**b**) Slip distance of fault after slipping.

**Fig. 6:**
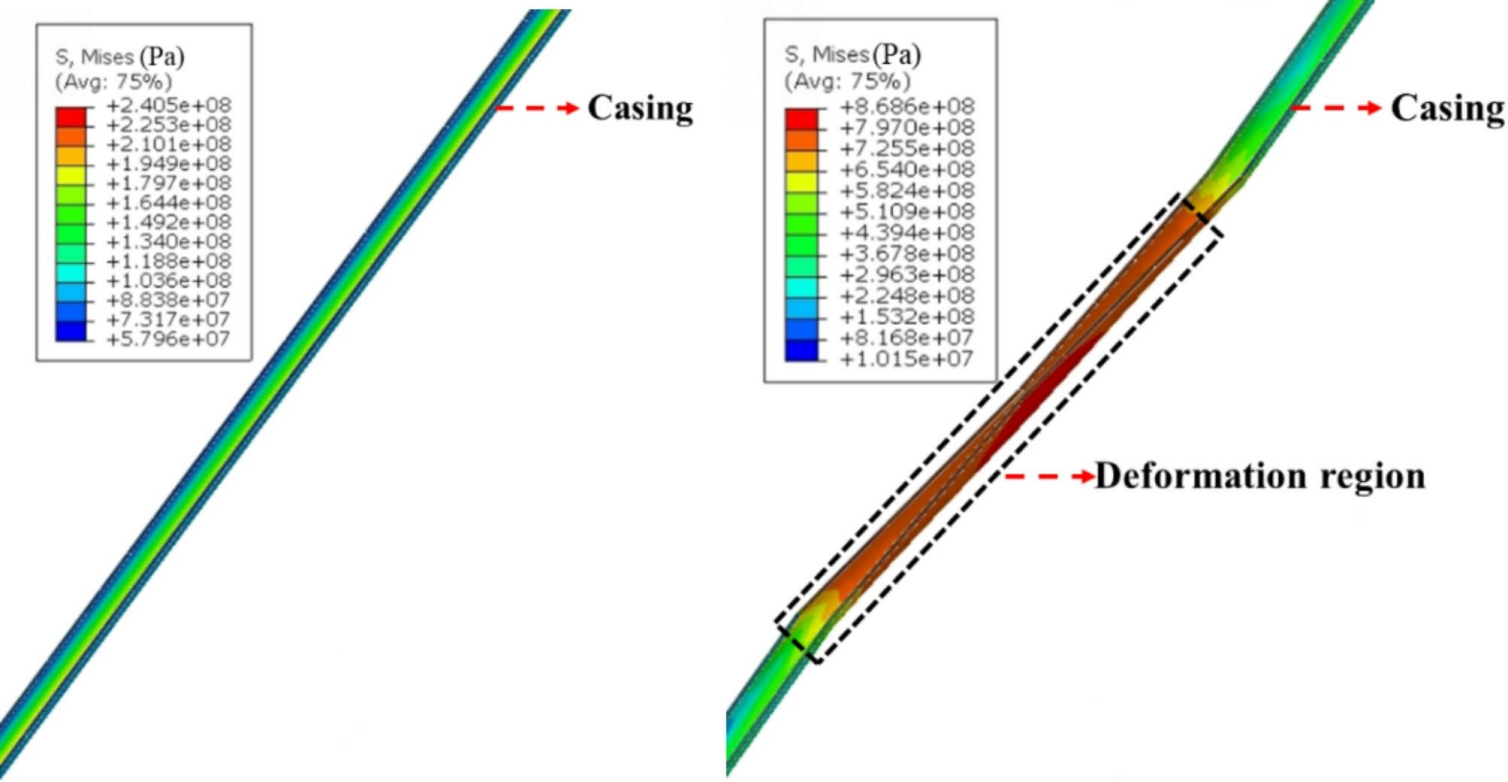
Characteristics of casing deformation. (**a**) Casing morphology before slipping (**b**) Casing morphology after slipping.

The model presented in Sect. 3 was utilized to compute the stress and deformation of the casing as it traverses the fault. The minimum fluid pressure required to activate the fault is 46 MPa, which is less than the actual fluid pressure of 65 MPa. Figures 5 and 6 display the slip distance of the fault and the morphology of casing deformation. It is observed that fault slip occurs under the influence of fluid pressure, leading to casing shear deformation near the fault due to the interaction between the fault and the casing. The shape of the casing is S-shaped following a fault slip. The fault’s maximum slip distance increased from 0 to 25 mm, while the casing’s maximum Mises stress rose from 241 MPa to 869 MPa following the occurrence of slipping.

**Fig. 7:**
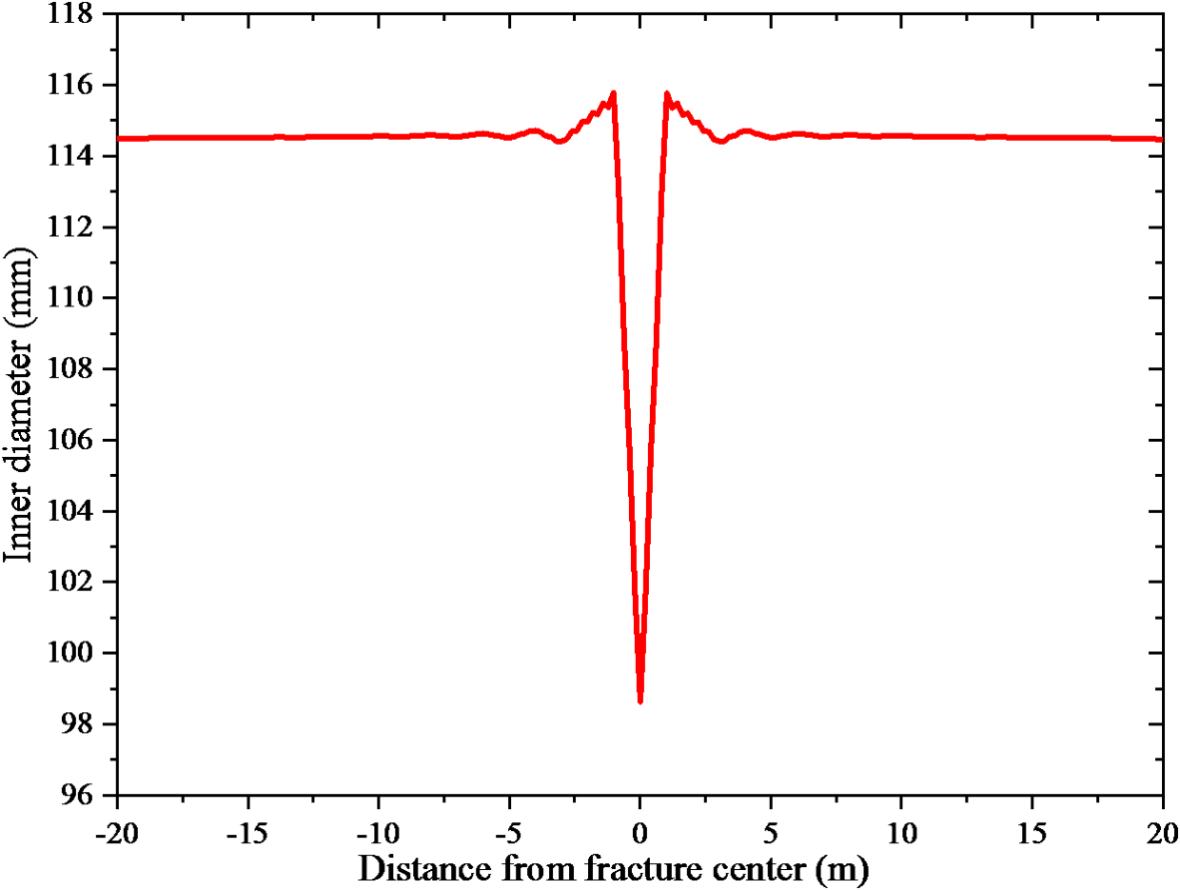
Casing deformation amount.

Figure 7 displays the inner diameter of the casing throughout the wellbore. Figure 7 illustrates that the inner diameter of the casing remains relatively consistent at a considerable distance from the fault. However, as the distance from the fault decreases, the casing’s inner diameter typically increases. The deformation of the casing is primarily localized within a distance of 1 m from the fault, reaching a maximum value of 15.7 mm. In addition, the fault exerts simultaneous radial and axial extrusion on the casing.

**Fig. 8:**
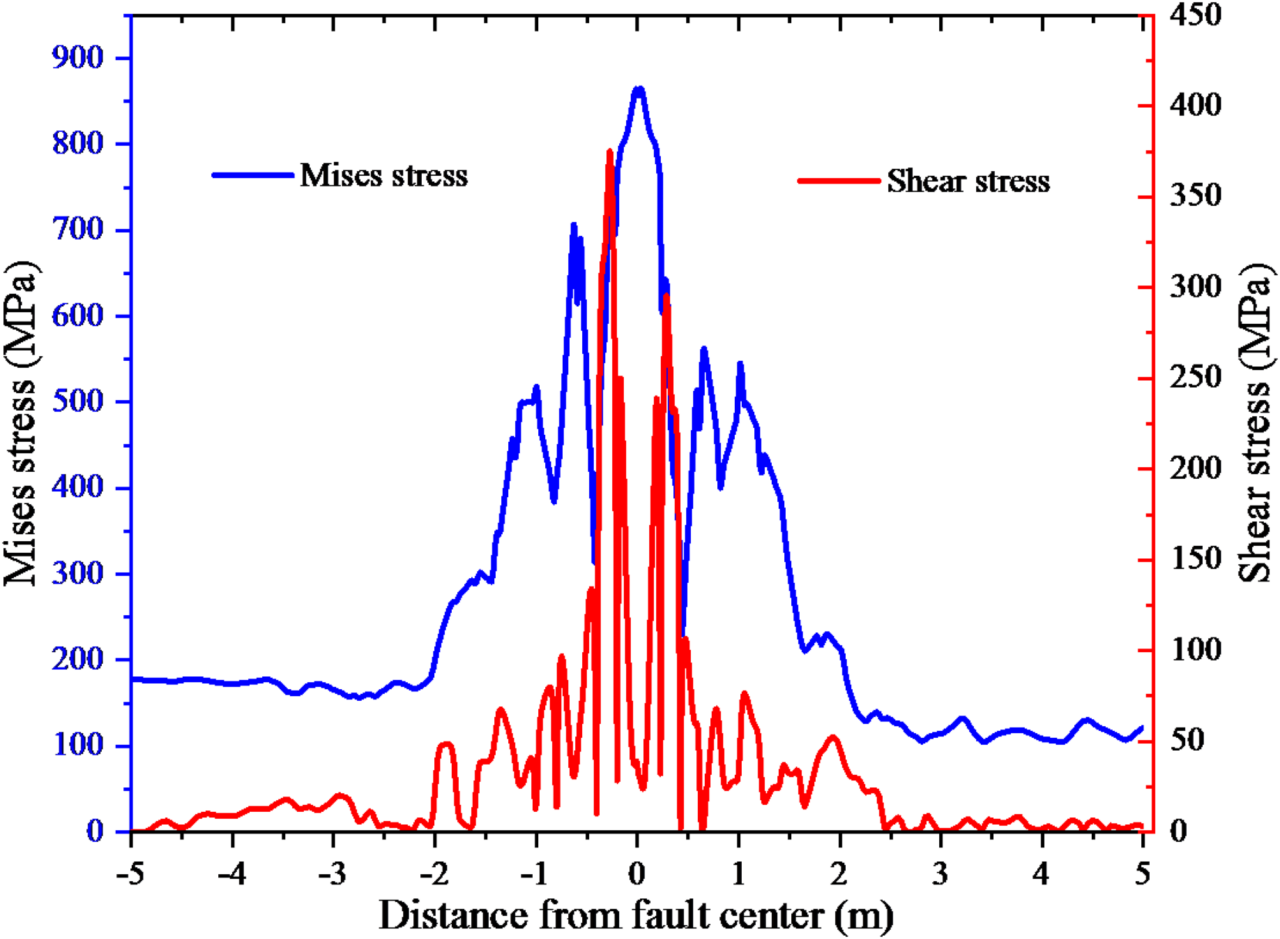
Casing shear stress and Mises stress.

The casing shear stress and Mises stress along the axial direction are shown in Fig. 8. Figure 8 illustrates the nearly symmetrical distribution of shear stress and Mises stress on the casing along the fault, owing to a specific angle between the casing and the fault. The casing exhibits notable stress concentration in the vicinity of the fault, where the maximum Mises stress and shear stress values reach 869 MPa and 375 MPa, respectively. Consequently, this specific region is highly susceptible to casing failure. As the distance from the fault decreases, the values of both variables increase. The increase is very small when the distance from the fault is far away, but increases sharply within 1 m from the fault.

Taking P110 casing as an example, its shear strength is 431 MPa and yield strength is 758 MPa. Based on the fourth strength theory, the casing is considered to have experienced yielding when its deformation reaches 15.7 mm. However, it is important to note that despite this deformation, the casing remains structurally intact and does not exhibit any signs of fracture. Based on the third strength theory, it is observed that the shear stress resulting from the fault slip is lower than the shear strength exhibited by the casing. Consequently, the casing remains intact without experiencing shear failure. Casing failure modes encompass shear failure, tensile failure, buckling failure and extruding failure. In fault slip scenarios, the primary mode of casing failure shifts to shear failure, thereby necessitating the application of the third strength theory for more suitable analysis and assessment.

### Coping measures for casing shear deformation

Casing shear deformation is significantly influenced by geological conditions, in-situ stress, and rock properties. Previous research has demonstrated that mitigating casing shear deformation resulting from formation slip can be achieved through non-cementing operations, lowering fracturing operation pressure, using temporary plugging fracturing technology, and optimizing wellbore trajectory^29,53–55^. By integrating 3D seismic data and formation parameters, the geomechanical model established in this study enables the prediction of casing shear deformation. This model can aid in selecting the area with small deformation for good placement and determining suitable fracturing parameters during initial wellbore trajectory design and subsequent fracturing operation.

#### Effect of fluid pressure within the fault and wellbore trajectory on casing deformation

**Fig. 9:**
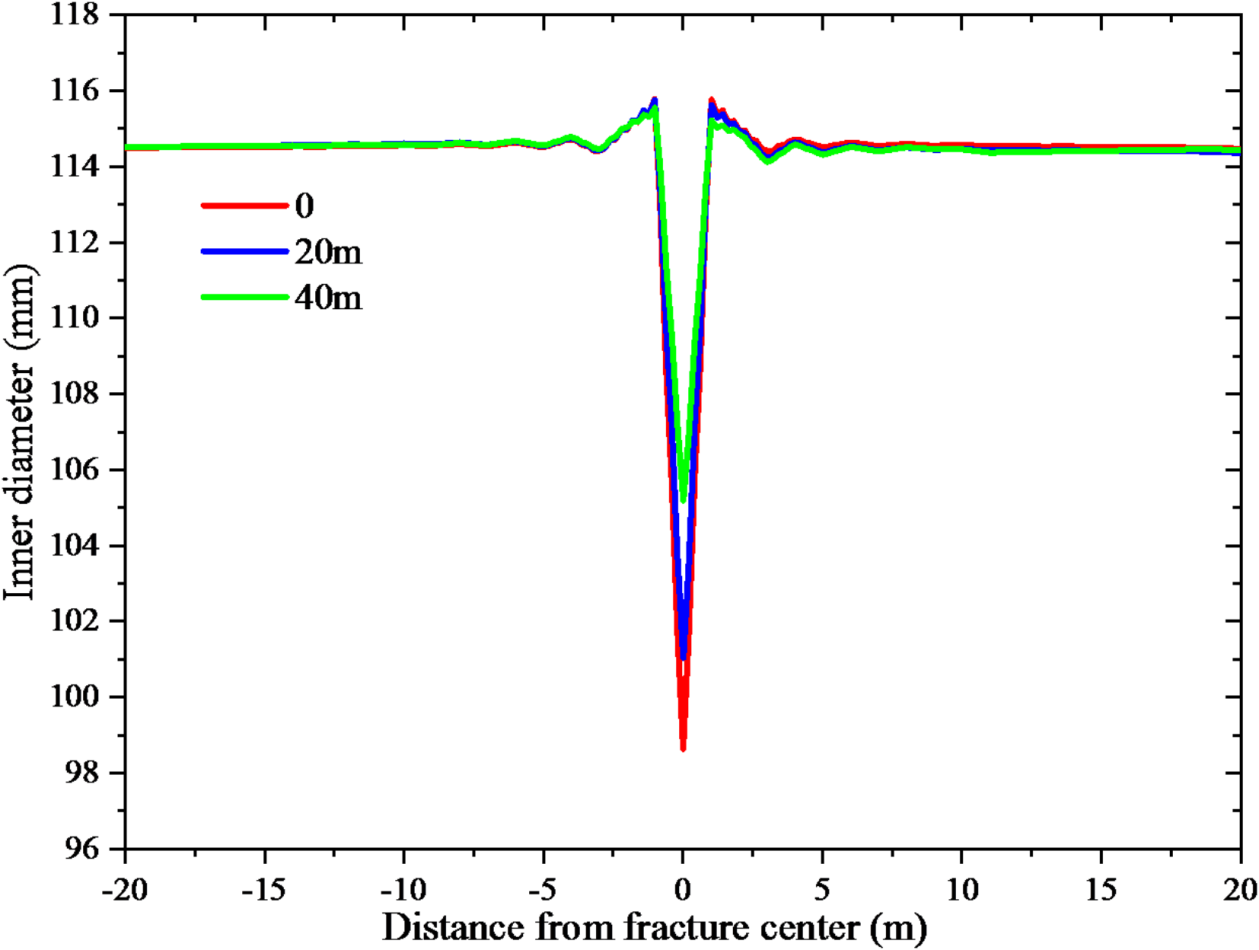
The effect of distance on casing deformation.

**Fig. 10:**
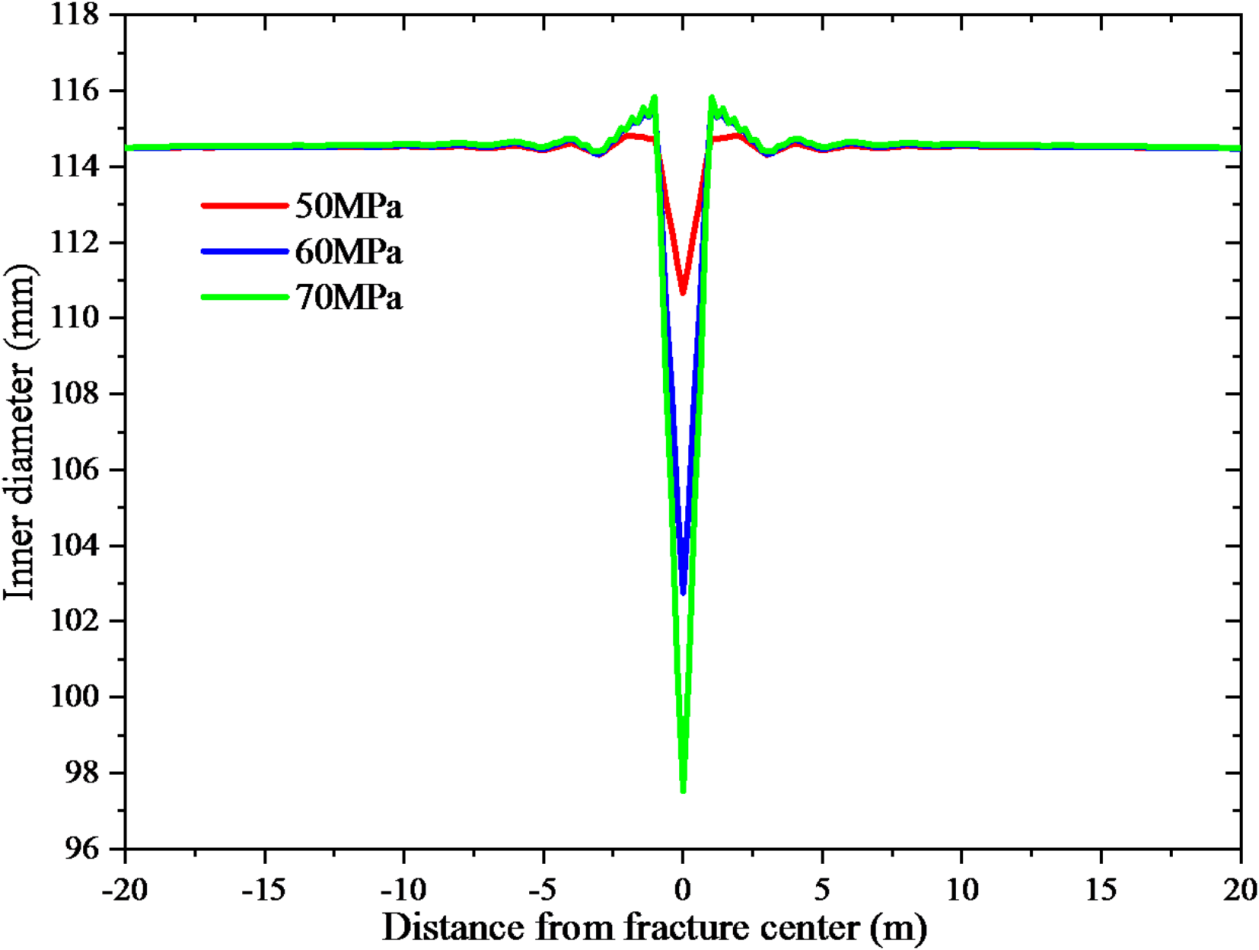
The effect of fluid pressure on casing deformation.

In order to address the issue of casing shear deformation, an investigation was conducted to analyze the individual effects of wellbore trajectory and fracturing parameters on casing deformation. In the process of drilling design, it is imperative to minimize contact with faults by carefully planning the trajectory of the well. If it cannot be avoided, the well trajectory should be arranged in the place far away from the fault center. The study examined three scenarios where the fault center was located at distances of 0, 20, and 40 m from the wellbore. The resulting casing deformation can be observed in Fig. 9. The casing deformation measurements were obtained at distances of 0, 20, and 40 m between the wellbore and fault center. The corresponding values for casing deformation were recorded as 15.7, 13.3, and 9.1 mm, respectively. As the distance from the fault center increases, the casing deformation decreases. In addition, the fluid pressure of 50, 60 and 70 MPa are set to analyze the influence of different operation pressures on casing deformation during fracturing. Figure 10 displays the casing deformation. The casing deformation measurements were obtained at fluid pressure levels of 50, 60, and 70 MPa, resulting in corresponding deformations of 3.6, 11.5, and 16.8 mm, respectively. As the fluid pressure increases, there is a corresponding increase in casing deformation.

#### Effect of casing and cement

**Fig. 11:**
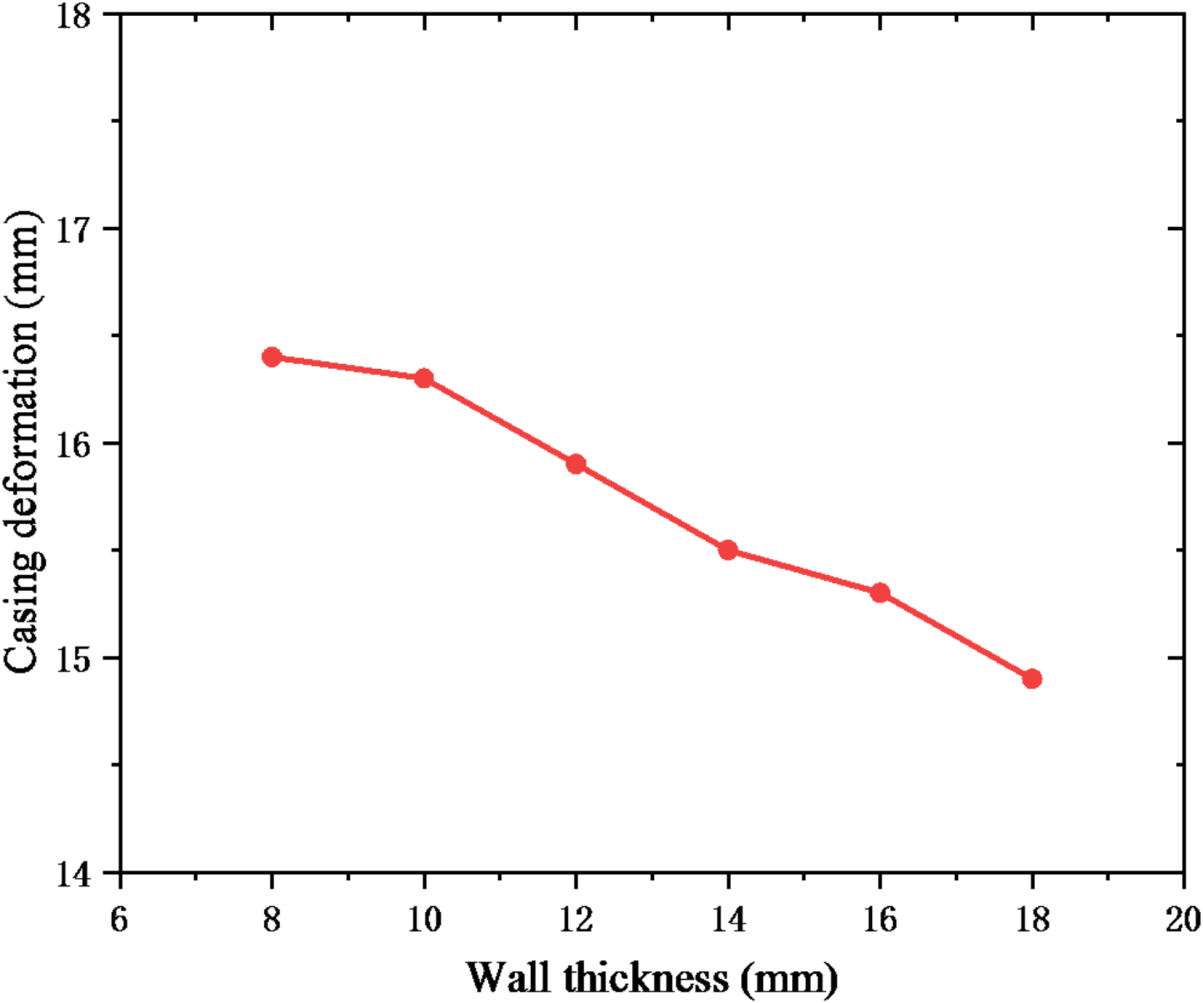
The effect of casing wall thickness on casing deformation.

**Fig. 12:**
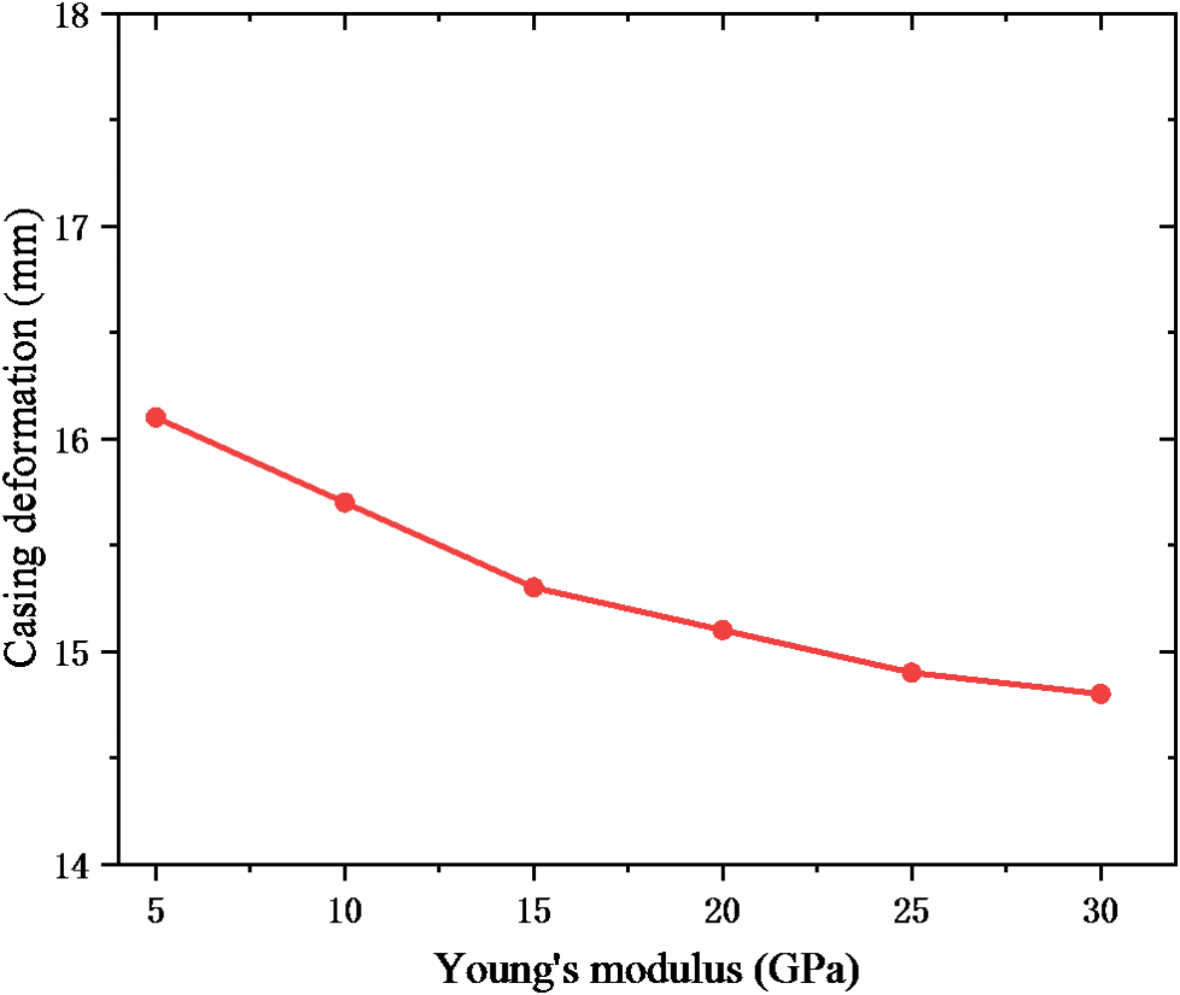
The effect of Young’s modulus on casing deformation.

Increasing the casing wall thickness is a common measure to enhance the shear resistance of the casing. The casing wall thickness was set to 8, 10, 12, 14, 16 and 18 mm, respectively. Figure 11 shows the reduction of casing inner diameter under different casing wall thicknesses. With the continuous increase of casing wall thickness, casing deformation slightly decreases, and the casing deformation decreased from 16.4 mm at the wall thickness of 8 mm to 14.9 mm at the wall thickness of 18 mm. The cement sheath plays the role of protecting the casing, which can alleviate the casing load. Therefore, the Young’s modulus of cement sheath was set to 5, 10, 15, 20, 25, and 30 GPa, respectively. The casing deformation is shown in Fig. 12. The casing deformation shows a downward trend under the continuous increase of Young’s modulus, but the change value of casing deformation is only 1.3 mm. Therefore, increasing the casing wall thickness and Young’s modulus of cement sheath does not significantly solve the casing shear deformation.

#### A novel approach to designing casing strength

The casing strength design method commonly employed necessitates that the casing yield strength exceeds the casing stress induced by external loads. The primary cause of casing failure resulting from fault slip is attributed to casing shear deformation, which significantly impacts the proper functioning of the fracturing tool. Therefore, it is an effective method to consider the casing shear deformation during casing design. Based on the findings above, it has been determined that the Mises stress criterion does not satisfy the necessary criteria for casing strength design in practical applications. A novel approach has been developed to enhance the casing strength design method by incorporating casing deformation. This newly proposed method serves as a valuable supplement to the current casing strength design approach. When the casing inner diameter is less than the maximum outer diameter of downhole tool, the wellbore integrity cannot ensure the normal fracturing operation, and the casing failure occurs in a specific form. In order to achieve optimal casing inner diameter, it is crucial to limit the casing deformation to a value that is below the maximum allowable deformation specified in the casing design. In the interim, it is imperative that the casing shear stress remains below the casing shear strength. The maximum allowable deformation of the casing is determined according to the inner diameter of casing and the outer diameter of downhole tool. The geological and engineering parameters are input into the model established to calculate the casing deformation. If the calculated deformation is found to be below the maximum allowable deformation for the casing, and if the initial design is deemed reasonable, then no further action is required. However, if either of these conditions are not met, it is necessary to make adjustments to the relevant engineering parameters.

## Field case analysis

**Fig. 13:**
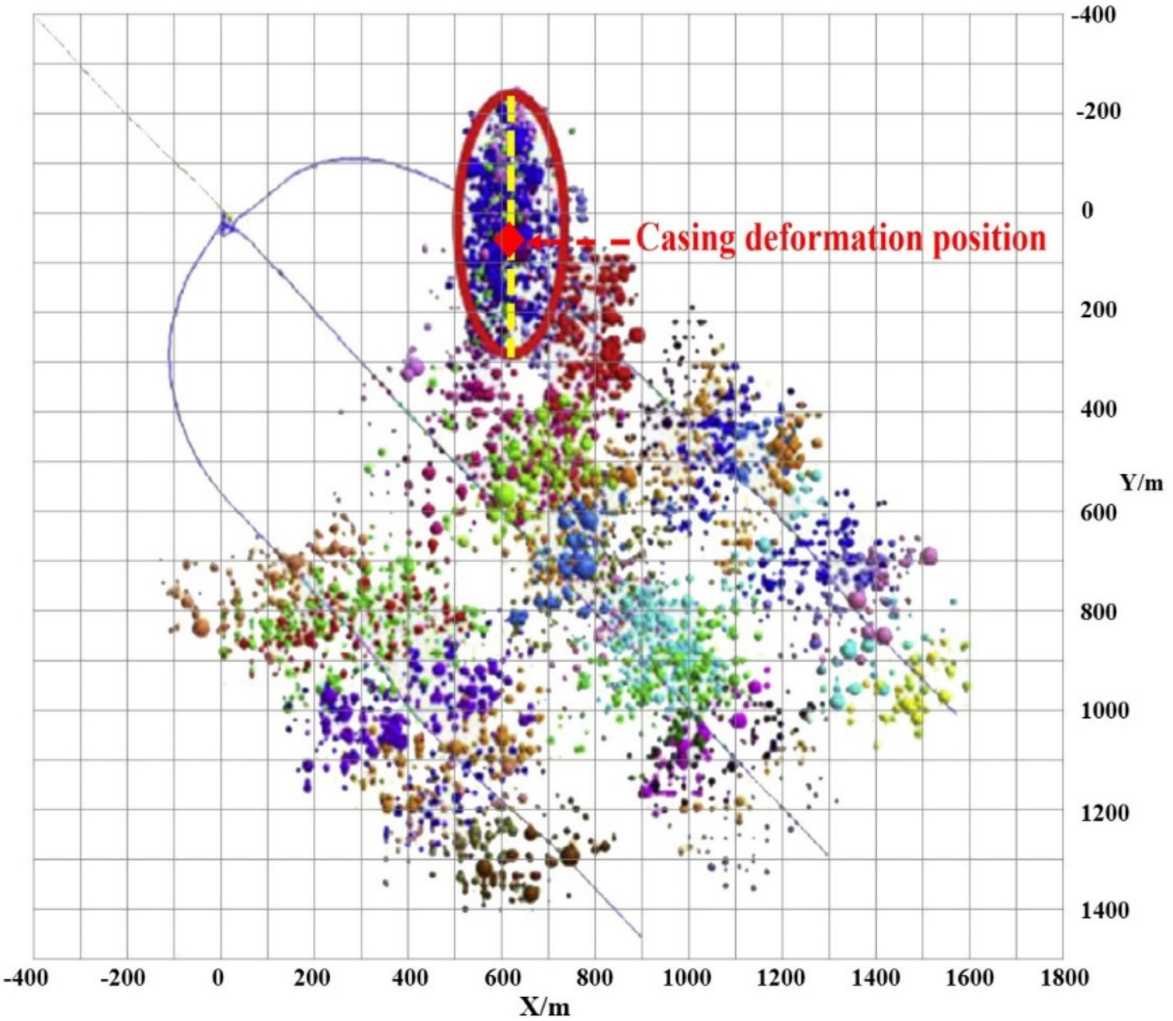
Distribution of microseismic events.

**Fig. 14:**
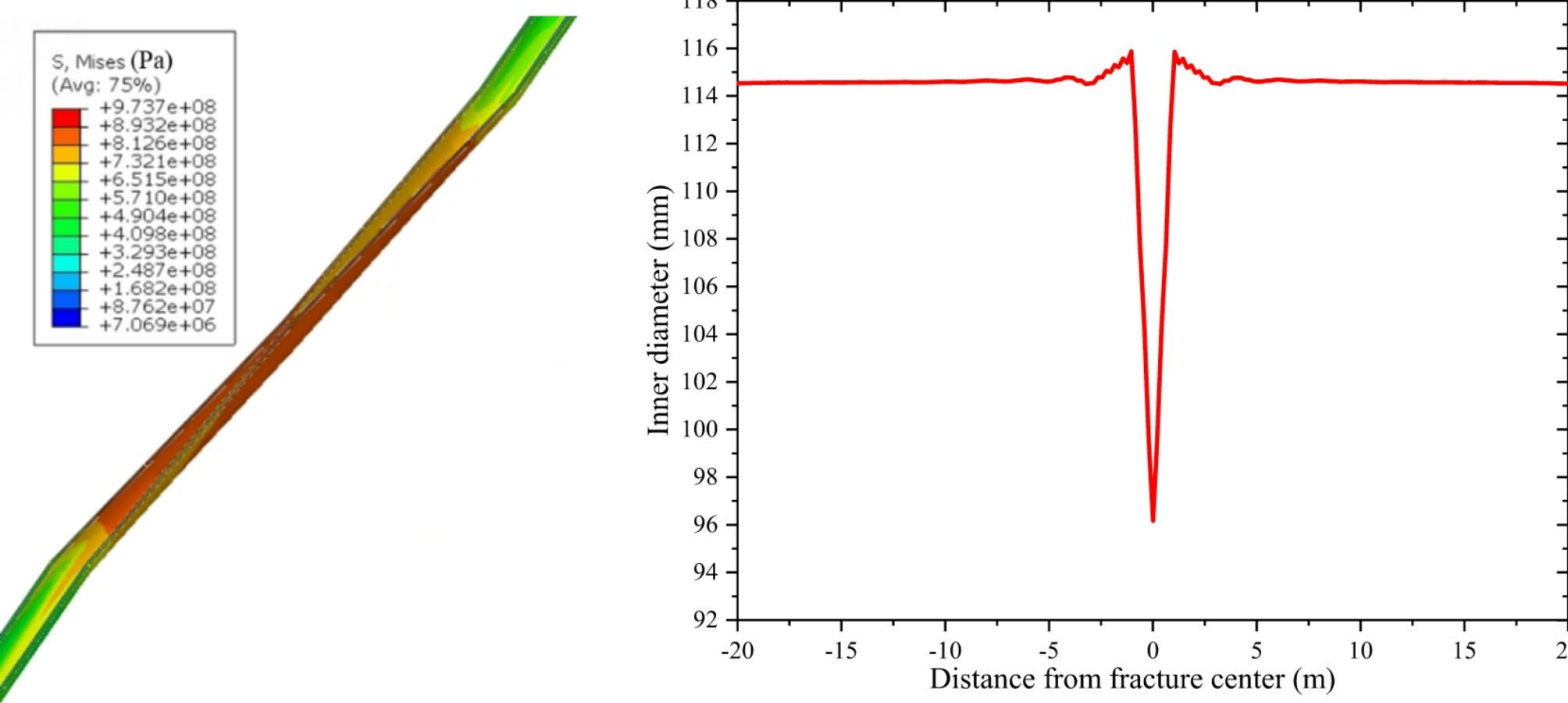
Casing deformation of W204HX. (**a**) Casing deformation morphology of W204HX (**b**) Casing deformation amount of W204HX.

The Well W204HX is a horizontal well with casing deformation in WYSGF. The horizontal section length of this well is 1435 m, and the casing of this well experienced deformation at a depth of 4155 m after the 12th fracturing stage was completed. The P110 casing was used with a yield strength of 758 MPa. The initial inner diameter of the casing is 114.3 mm, while the deformation of the casing measures 20.06 mm. Microseismic monitoring revealed an oblique microseismic signal in relation to the wellbore, indicating the activation of the fault. The fault has an approximate diameter of 500 m and is positioned at a 45-degree angle from the wellbore. The deformation of the casing takes place 50 m away from the center of the fault, as depicted in Fig. 13. The vertical stress, maximum horizontal stress, and minimum horizontal stress are 42, 48 and 29 MPa, respectively. The fluid pressure is 47 MPa. The casing deformation was calculated using the established mode as described in Sect. 3. The maximum casing deformation, as depicted in Fig. 14, is determined to be 18.13 mm. The model’s calculated results exhibit a minor deviation from the actual value, thereby confirming the model’s reliability.

## Conclusion

This paper presents an analysis of the seismicity and casing deformation observed in the shale gas field located in the Sichuan basin. The present research provides a summary of the tectonic characteristics and stress fields observed in various shale gas fields. Additionally, it explores the relationship between these factors and casing deformation. In addition, a three-dimensional geomechanical model was developed to simulate the casing deformation resulting from fault slip. The following are the conclusions:

1. The WYSGF, CNSGF and ZTSGF formations in the Sichuan basin demonstrate notable seismic activity and a substantial rate of casing deformation. The aforementioned areas are primarily located within the slopes of anticlines and synclines, which are known for experiencing significant tectonic stress, notable differences in horizontal stress, and the presence of well-developed faults. In contrast, the FLSGF within the Sichuan basin predominantly occupies a tectonic region characterized by a combination of tensile and compressive forces. This region exhibits stress release, minimal differences in horizontal stress, and a relatively low rate of casing deformation.
2. The process of hydraulic fracturing results in significant casing deformation and casing stress when faults are triggered. Casing deformation predominantly manifests within a proximity of 1 m from the fault. One approach to mitigating casing deformation involves the reduction of operation pressure during fracturing and the implementation of wellbore management strategies when crossing fault locations. By decreasing the pressure exerted during the fracturing process and carefully managing the positioning of the wellbore in relation to fault, the potential for casing deformation can be minimized.
3. A proposed casing strength design method is presented in this research, which aims to address casing shear deformation during hydraulic fracturing. This method is developed based on the analysis of casing deformation and stress. The methodology relies on the established stress strength design principle and utilizes the casing deformation amount as the primary control target. The prediction and control of casing deformation can be achieved during the casing design stage.

## Data Availability

The data analysed during the current study are not publicly available due to data privacy, but are available from the corresponding author on reasonable request.
